# Long Term Monitoring in Switzerland Reveals That *Adalia bipunctata* Strongly Declines in Response to *Harmonia axyridis* Invasion

**DOI:** 10.3390/insects11120883

**Published:** 2020-12-12

**Authors:** Marc Kenis, Saidou Nacambo, Johan Van Vlaenderen, Renate Zindel, René Eschen

**Affiliations:** 1 CABI, 1 Rue des Grillons, 2800 Delémont, Switzerland; s.nacambo@cabi.org (S.N.); j.vanvlaenderen@gmail.com (J.V.V.); renatezindel@gmail.com (R.Z.); r.eschen@cabi.org (R.E.); 2 Department of Biology, University of Fribourg, 1700 Fribourg, Switzerland

**Keywords:** *Adalia bipunctata*, Coccinellidae, *Harmonia axyridis*, invasive species, ladybirds

## Abstract

**Simple Summary:**

The harlequin ladybird, *Harmonia axyridis*, is an Asian species that has invaded Europe and other continents, where it is suspected to cause the decline of native ladybirds through competition and predation. In north-western Switzerland, ladybirds were monitored for 11 years in four habitats (broadleaved hedges, meadows, pine and spruce stands) to assess the decline of native ladybirds following the invasion of the harlequin ladybird. These surveys showed that, on broadleaved hedges, the harlequin ladybird quickly became the most abundant species, representing 60–80% of all specimens collected in this habitat. One species, the two-spot ladybird, *Adalia bipunctata*, almost disappeared during this period, whereas it was the most abundant ladybird on broadleaved trees and shrubs when this study started. The other native species did not show any clear sign of decline. The harlequin ladybird was the second most abundant species in pine stands, and was not abundant in meadows and in spruce stands. The total number of ladybirds feeding on aphids did not decline during this period, suggesting that the arrival of the harlequin ladybird did not affect the predation pressure on aphids. Nevertheless, the severe decline of the two-spot ladybird deserves further investigations.

**Abstract:**

A long-term monitoring was conducted at 40 sites in four different habitats in north-western Switzerland to observe changes in populations of native ladybirds, following the invasion of the Asian harlequin ladybird, *Harmonia axyridis*. From 2006 to 2017, the same trees and meadows were sampled at least seven times per year using standard protocols. On 15 broadleaved hedges, *H. axyridis* quickly became the dominant species, representing 60 to 80% of adult ladybirds collected. It was second in abundance at five pine (*Pinus sylvestris*) stands and was a minor component of the ladybird complex at five spruce (*Picea abies*) stands and in 15 meadows. This survey revealed the severe decline of *Adalia bipunctata*, which was the most abundant native ladybird on broadleaved trees in 2006–2009 and has almost disappeared since 2010. So far, other native ladybirds do not seem to decline significantly, including species occupying the same ecological niches as *H. axyridis*. The total number of aphidophagous ladybirds did not decline either, suggesting that the biological control function of ladybirds on aphids living in these habitats has not been affected by the arrival of *H. axyridis*. Recommendations are given to further assess the impact of *H. axyridis* on native ladybirds and aphids.

## 1. Introduction

The harlequin ladybird, *Harmonia axyridis* (Pallas) (Coleoptera: Coccinellidae), is a polyphagous predator native to Central and East Asia, which has been used as a biological control agent against aphids in North America and Europe, in greenhouses as well as in outdoor crops. In North America, it has been considered established in the wild since 1988, and very quickly became the dominant ladybird in many ecosystems [1]. In Europe, it has been tested since 1982, and was first marketed in 1995 [2]. Since populations were first observed in the wild in 1999 in Germany, its abundance has increased exponentially, and the species is now established in almost all European countries [3]. In Switzerland, a first individual was found in Basel in 2004 and the establishment of the species was confirmed in 2006 [4]. It is now found throughout the country except in high elevation areas [5].

The invasion of *H. axyridis* has a number of negative effects [3]. It is a human nuisance when it aggregates in buildings in autumn and can taint wine when harvested and crushed with grapes. However, most concern so far has been caused by its impact on biodiversity. Due to its predatory and competitive abilities, *H. axyridis* may affect many native species, including non-pest aphids and aphidophagous insects. In particular, native ladybirds may suffer from competition for resources and intra-guild predation (IGP) on larvae and eggs ([3] and references therein). In North America, several studies showed that *H. axyridis* has displaced native ladybirds [6,7,8]. Similar observations were made in Chile, where it is also invasive [9]. In Europe, first analyses made a few years after the establishment of *H. axyridis* in UK, Belgium and Switzerland suggested that several native ladybird species had started declining [10,11]. In a risk assessment based on the likelihood that the assessed native species encounters *H. axyridis* in the field, the hazard of competition for food, and the IGP hazard, Kenis et al. [12] stated that four European species were particularly at risk, i.e., *Adalia bipunctata* (L.)*, Adalia decempunctata* (L.)*, Oenopia conglobata* (L.) and *Calvia decemguttata* (L.). These are species that, as *H. axyridis*, are found mainly on deciduous trees, are primarily aphidophagous and have immature stages which are highly vulnerable to IGP by *H. axyridis*. These studies and others emphasized the need for further field assessments of native ladybirds’ decline in Europe but, since ladybird populations are known to naturally fluctuate from year to year [13,14], it is essential that such assessments are based on long-term studies. Brown and Roy [15] provided population changes at four sites in England over an 11-year period (2006–2016) following invasion by *H. axyridis*. Their survey focused on three plants: lime trees (*Tilia* x *europea*), pine (*Pinus sylvestris*) and nettle (*Urtica dioica*). While *H. axyridis* was over three times more abundant than the second commonest species, it only dominated at the lime sites and only one native species, *A. bipunctata*, significantly declined.

Here, we present the results of another long-term survey conducted at 40 sites in Switzerland in the period 2006–2017, to observe changes in populations of native ladybirds before, during and after the arrival of *H. axyridis* and assess their potential decline.

## 2. Materials and Methods

A long-term field trial was set up in north-western Switzerland in the cantons Jura, Basel-Landschaft, Basel-Stadt and Aargau, based on 40 permanent sites established within a 40 km distance from the town of Delémont (Table S1 in Supplementary file). Thirty sites were selected in 2006: 15 “meadow sites” consisting of managed and unmanaged meadows, along roads, sampled by sweeping 60 times through the vegetation on a transect of 30 m with a 60 cm-diameter sweep net; 15 “broadleaved hedges” consisting of clearly delimited, ca. 50 m long planted hedgerows, semi-natural hedges or forest hedges of mixed broadleaved trees and shrubs, sampled by beating all branches up to a height of 2.5 m with a stick and collecting beetles falling down on a white, rectangular 90 × 125 cm beating tray. In 2007, 10 “conifer sites” were selected, all in the Jura, except one in Basel-Landschaft. They consisted of 4 to 10 preselected individual pine (*Pinus sylvestris*, 5 sites), and spruce (*Picea Abies*, 5 sites) trees, of which all branches up to 2.5 m were beaten above the same beating tray. All sites were chosen haphazardly. The same sites were sampled seven to ten times per year per site, from mid-April to late September from 2006 (2007 for the conifers) until 2013. No sampling was conducted in 2014 because of budget constraints. In the period 2015–2017, similar collections were made at the same sites, except that one site of broadleaved hedges had disappeared and was replaced by another in the same region. Five meadow sites were also replaced by other, very similar meadows.

Adult ladybirds were identified to species on-site using various identification keys [16,17,18] and all ladybirds (adults and larvae) were replaced on the branches or in the meadow after identification. Only “true” ladybirds, i.e., those belonging to the sub-families Coccinellinae, Chilocorinae and Epilachninae were included in the study, because they are easier to identify in the field than the small Coccidulinae and Scymninae [18]. The amount of aphidophagous ladybird adults obtained per collection on broadleaved hedges was assessed, and the ladybird species were classified as mainly aphidophagous, based on Kenis et al. [12].

## 3. Results

**Figure 1:**
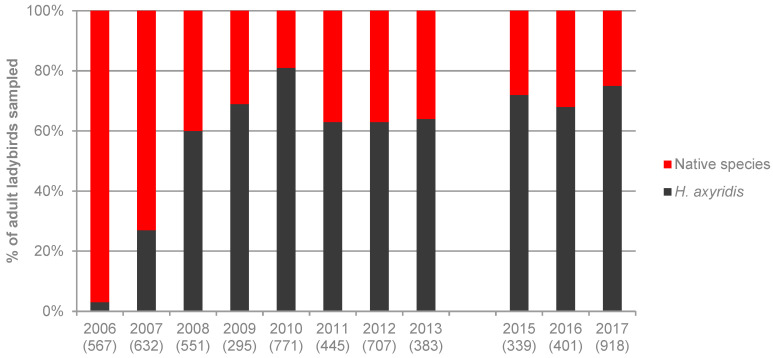
Percentage of *Harmonia axyridis* and native species at the 15 broadleaved hedges from 2006 to 2017. Numbers between parentheses indicate the number of adult ladybirds collected.

**Figure 2:**
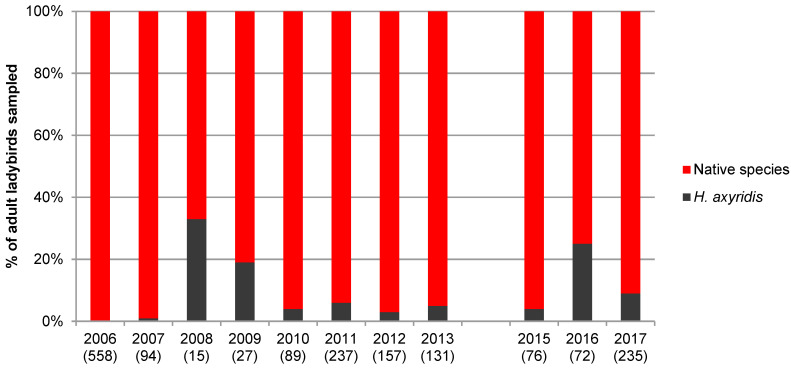
Percentage of *Harmonia axyridis* and native species in the 15 meadows from 2006 to 2017. Numbers between parentheses indicate the number of adult ladybirds collected.

**Figure 3:**
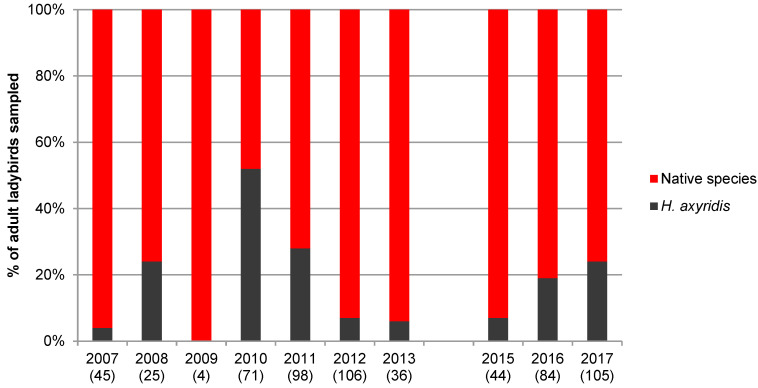
Percentage of *Harmonia axyridis* and native species at the five pine sites from 2007 to 2017. Numbers between parentheses indicate the number of adult ladybirds collected.

**Figure 4:**
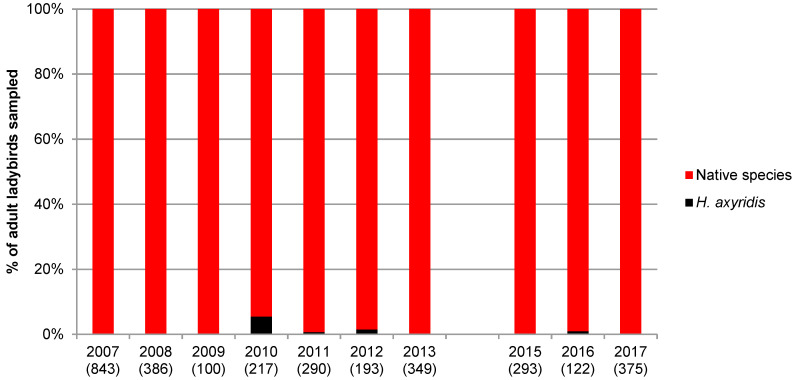
Percentage of *Harmonia axyridis* and native species at the five spruce sites from 2007 to 2017. Numbers between parenthesis indicate the number of adult ladybirds collected.

A total of 10,248 adults belonging to 25 species were collected during this study. Figure 1, Figure 2, Figure 3 and Figure 4 show the proportion of *H. axyridis* in the four habitats since the start of sampling in 2006 and 2007 and Table 1, Table 2, Table 3 and Table 4 give details of the species collected.

**Figure 5:**
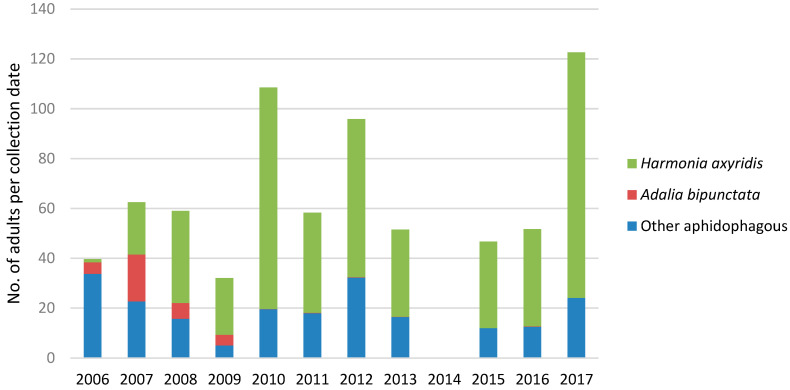
Number of adults of *Harmonia axyridis*, *Adalia bipunctata* and other aphidophagous ladybird species collected per collection date on 15 broadleaved hedges from 2006 to 2017.

On broadleaved hedges, *H. axyridis* was present in low numbers in 2006, at two of the 15 sites. In 2007, it already represented 27% of all ladybird beetles and from 2008 to 2017, its proportion varied between 60 and 80% (Figure 1). A total of 21 native species were collected on the hedges (Table 1). Although the number of specimens collected per species varied between years, only one species, *A. bipunctata*, clearly declined during this period (Table 1, Figure 5). It was the main native species from 2006 to 2009 and, since 2010, not more than one specimen per year was collected. Other abundant species included *A. decempunctata* (13.5% of all ladybirds), *Propylea quatuordecimpunctata* (L.) (8.9%) and *Calvia quatuordecimguttata* (L.) (5.5%). Two species, *Chilocorus bipustulatus* (L.) and *Chilocorus renipustulatus* (Scriba) were abundant only in 2007, but this was mainly at one site, and their abundance was related to a local outbreak of a scale insect. The total number of aphidophagous ladybirds was highly variable among years, but there was no sign of decline, with the highest number of specimens collected in 2017 (Figure 5).

In meadows, the proportion of *H. axyridis* has remained low since its arrival in Switzerland (Figure 2 and Table 2). Since *H. axyridis* became abundant in the region in 2008, it represented 3 to 33% of all ladybirds collected in meadows. Sixteen native species were collected, the three most abundant being *Tytthaspis sedecimpunctata* (L.) (41.8%), *P. quatuordecimpunctata* (26.6%) and *Coccinella septempunctata* L. (12.6%).

On conifers, since the start of the surveys, *H. axyridis* has been more abundant on pine than on spruce. The proportion of *H. axyridis* on pine varied from 0% in 2009 to 52% in 2010 (Figure 3 and Table 3). These strong variations are also explained by the low and fluctuating numbers of ladybirds collected on pine (four to 114 individuals per year). Fifteen native species were found on pine and the most abundant species was *Harmonia quadripunctata* Pontoppidan (26.3%) followed by *H. axyridis* (20.2%) and *C. septempunctata* (16.4%).

Ladybirds were much more abundant on spruce (100 to 843 individuals per year), but the proportion of *H. axyridis* generally did not exceed 1% (Figure 4 and Table 4). On spruce, the dominant ladybird was by far *Aphidecta obliterata* (L.) (76.3%), but 15 native species were collected, including significant numbers of *C. septempunctata* (13.9%), which was the most abundant species at one site throughout the period.

## 4. Discussion

Our long-term survey of ladybirds in north-western Switzerland showed that, on broadleaved trees and shrubs, *H. axyridis* has become by far the most abundant species just a few years after its arrival in Switzerland. Since then, it started to represent between 60 and 80% of all adult ladybirds. Similar levels of dominance on broadleaved trees were also found recently in other European countries such as England [15], Czech Republic [19] and Italy [20].

Although it is known that ladybird populations can vary greatly from year to year [13] (see also Table 1, Table 2, Table 3 and Table 4 for variations observed in some ladybirds, e.g., *C. septempunctata and T. sedecimpunctata*), the fact that *A. bipunctata* has almost disappeared from our records since 2010 strongly suggests that this decline in populations is not due to natural fluctuations in populations but more probably to the presence of *H. axyridis*. *Adalia bipunctata* used to be the most abundant species in this habitat in Switzerland [4] and other European countries, e.g., [19,20,21,22]. Another 11-year study in England also showed that *A. bipunctata* was the only species to decline significantly after the arrival of *H. axyridis* [15]. Signs of recent decline were also found in other countries such as Czech Republic [19], Italy [20] and Belgium [11]. In the latter country, the decline of *A. bipunctata* warrants red listing as a vulnerable species [23]. *Adalia bipunctata*, a Holarctic species, is also one of the species that shows the strongest decline in Eastern North America, following the invasion of *H. axyridis* and three other exotic ladybird species [8]. However, in none of these European and American studies was the decline as strong as in our surveys in north-western Switzerland. For example, in England, *A. bipunctata* abundance in 2016 was reduced to approximately 16% of the total from the first surveys in 2006 [15]. Although it has almost disappeared from north-western Switzerland, opportunistic surveys showed that it is still more abundant in other regions, such as in the Valais, southern Switzerland, particularly on deciduous trees in urban settings, perhaps because of the abundance of resources over a long period of the year, which limits intra-guild competition [5].

The decline of *A. bipunctata* following the invasion of *H. axyridis* was expected. The ecological niches of *A. bipunctata* and *H. axyridis* greatly overlap [12,21] and several laboratory studies showed that *A. bipunctata* does not have the same defenses, as other ladybirds to thwart predation by *H. axyridis* e.g., [24,25,26]. For example, eggs and larvae of *C. quatuordecimguttata* are chemically protected against IGP by *H. axyridis*, whereas species such as *Anatis ocellata* (L.) or *C. septempunctata* have mechanical defense mechanisms. However, three other species (*A. decempunctata*, *C. decemguttata* and *O. conglobata*) also lack defense mechanisms, and have been indicated as potentially at risk in a risk analysis [12]. Yet, they do not seem to decline in north-western Switzerland, at least for now. *Adalia decempunctata* is now the most abundant native species on broadleaved trees and shrubs. The other two species are less common but still present. In particular, the rare *C. decemguttata* is regularly monitored on lime trees in the Swiss Jura, and populations do not show any sign of decline [5]. Honek et al. [19] even state that the species is increasing in the Czech Republic despite *H. axyridis*. The total number of ladybird adults collected in our surveys on broadleaved trees varied quite strongly from year to year, but 2017 was the most successful year in terms of adults collected, suggesting that the biological control function of ladybirds on aphids feeding on broadleaved trees has not been affected by the arrival of *H. axyridis*. The same conclusion was drawn by Bahlai et al. [13] based on a long-term ladybird survey in Michigan, USA.

Early reports from Belgium and England already showed that *H. axyridis* can become rather abundant on herbaceous plants and conifers [10,21]. In our surveys it never became the dominant species in these habitats, in particular, on spruce, where it was almost absent. A long-term study [15] in England also observed that it was not dominant on pine and nettles, where it represented 11.4% (fourth most abundant species) and 5.3% (fifth) of all ladybirds, respectively. These results suggest that it does not represent a significant danger to native ladybirds that are found specifically on conifers and grasses. Nevertheless, the fact that *H. axyridis* represents 20.2% of all ladybirds on pine in north-western Switzerland prompts us to suggest further studies in this habitat. Furthermore, various studies, mostly from the Americas, showed that it can also become very abundant on annual crops, such as alfalfa fields in Chile [9,27] potato fields [28] or across agricultural landscapes [6,8,13] in USA. Thus, their impact on both aphids and aphidophagous insects in such habitats should also be investigated in Europe.

Following this and other studies on the decline of *A. bipunctata*, the following recommendations are made. Firstly, this database of ladybird abundance at 40 sites for 11 years is a unique tool to assess the impact of an invasive insect in the long term. It would be important to sample again at the same sites in a few years to verify that *H. axyridis* remains the dominant species, that the decline of *A. bipunctata* is confirmed, and that other native lady beetles are not declining. Sampling for at least 3–4 years would be essential to consider the effects of natural fluctuations in ladybird populations.

Secondly, the decline of *A. bipunctata* should be studied more comprehensively. A national or continental assessment of the situation of *A. bipunctata* would be desirable, since the ladybird does not decline equally everywhere. It would also be necessary to test and analyze, through field observations and laboratory experiments, the factors favoring or hampering the presence of *A. bipunctata*, as well as the mechanisms explaining why this species rather than another is declining. Laboratory studies have already shown that some species of ladybirds such as *C. quatuordecimguttata*. and *C. septempunctata* had chemical or physical defenses against *H. axyridis* [12,26,29], but other species, such as *A. decempunctata*, *C. decemguttata* and *O. conglobata*, also appear vulnerable and, yet, are not affected. It has also been suggested that the decline of native species following the invasion of *H. axyridis* could be due to exploitative or apparent competition through common natural enemies, such as predators, pathogens and parasitoids [30]. This possibility should be further investigated.

Thirdly, the effect of the invasion of *H. axyridis* on aphid populations is still poorly documented in Europe. Unverified information points to a decreasing trend, or at least a status-quo, in aphid-related agricultural problems, which would confirm that the arrival of *H. axyridis* did not alter the natural aphid control function of ladybirds by affecting native ladybird populations. However, it is not excluded that *H. axyridis* has a direct negative effect on native aphid populations, in particular aphids living on deciduous trees. Although it is difficult, long after the invasion of *H. axyridis*, to measure this effect accurately, it is possible that old and long-term data from aphid populations can be compared to new surveys carried out using the same protocols.

## 5. Conclusions

Our long-term monitoring of ladybird populations in north-western Switzerland clearly showed that *H. axyridis* quickly became the dominant species on broadleaved trees and shrubs just a few years after its arrival, but not yet on conifers and grasses. Only one native species, *A. bipunctata*, clearly declined following the invasion of *H. axyridis*, but this once dominant species almost disappeared from the region. The severe decline of *A. bipunctata* deserves further investigations.

## Figures and Tables

**Table 1:** Species and number of adult ladybirds collected at the 15 broadleaved hedges from 2006 to 2017. Numbers between parentheses indicate the number of collections. Ladybirds were not sampled in 2014.

	2006 (9–10)	2007 (8–9)	2008 (9)	2009 (9)	2010 (7)	2011 (7)	2012 (7)	2013 (7)	2015 (7)	2016 (7)	2017 (7)	Total
*Adalia bipunctata* (L.)	46	150	57	39	1	1	1	1	0	1	0	297
*Adalia decempunctata* (L.)	92	67	94	24	79	18	91	41	21	52	93	672
*Anatis ocellata* (L.)	1	1	0	0	0	0	1	0	0	0	0	3
*Aphidecta obliterata* (L.)	0	0	0	0	0	0	0	1	0	0	0	1
*Calvia quatuordecimguttata* (L.)	31	60	21	5	21	20	21	17	17	22	39	274
*Calvia decemguttata* (L.)	0	0	1	1	1	0	1	0	4	0	7	15
*Chilocorus bipustulatus* (L.)	0	42	2	0	1	1	0	0	0	0	3	49
*Chilocorus renipustulatus* (Scriba)	0	36	0	0	0	0	0	0	0	0	1	37
*Coccinella quinquepunctata* L.	1	0	0	0	0	0	0	0	0	0	0	1
*Coccinella septempunctata* L	23	10	1	2	7	46	46	7	15	1	1	159
*Exochomus quadripustulatus* (L.)	37	41	2	1	2	24	27	9	6	12	17	178
*Halizia sedecimguttata* (L.)	23	1	7	3	1	10	1	0	3	2	32	83
***Harmonia axyridis* (Pallas)**	**13**	**168**	**332**	**205**	**622**	**281**	**444**	**245**	**243**	**273**	**689**	**3515**
*Harmonia quadripunctata* Pontoppidan	2	2	2	0	0	1	0	0	0	0	0	7
*Henosepilachna argus* (Geoffroy)	0	0	0	0	0	0	1	1	1	0	0	3
*Hippodamia variegata* (Goeze)	2	1	0	0	0	0	0	0	0	0	1	4
*Myzia oblonguttata* (L.)	0	0	0	0	0	0	0	0	1	0	0	1
*Oenopia conglobata* (L.)	2	13	5	1	3	3	10	4	2	2	7	52
*Platynaspis luteorubra* (Goeze) *	14	2	1	0	0	0	5	2	0	0	0	24
*Propylea quatuordecimpunctata* (L.)	170	26	17	12	26	38	51	44	24	14	21	443
*Psyllobora vigintiduopunctata* (L.)	9	11	1	2	6	2	7	11	2	22	3	76
*Subcoccinella vigintiquatuorpunctata* (L.)	1	1	8	0	1	0	0	0	0	0	4	15
Total	467	632	551	295	771	445	707	383	339	401	918	4991

* In the field, *Platynaspis luteorubra* can be confused with Scymninae, a sub-family not covered in this study. Therefore, it cannot be ruled out that some specimens have been misidentified.

**Table 2:** Species and number of adult ladybirds collected in the 15 meadows from 2006 to 2017. Numbers between parentheses indicate the number of collections. Ladybirds were not sampled in 2014.

	2006 (9)	2007 (8–9)	2008 (9)	2009 (9)	2010 (7)	2011 (7)	2012 (7)	2013 (7)	2015 (7)	2016 (7)	2017 (7)	Total
*Adalia bipunctata*	0	4	2	6	0	0	0	0	0	0	0	12
*Adalia decempunctata*	1	0	1	3	0	2	0	0	0	4	4	15
*Anatis ocellata*	1	0	0	0	0	0	0	0	0	0	0	1
*Calvia quatuordecimguttata*	0	1	0	0	1	0	0	0	0	1	3	6
*Chilocorus renipustulatus*	0	1	0	0	0	0	0	0	0	0	0	1
*Coccinella quiquepunctata*	0	0	0	1	1	0	0	0	0	0	0	2
*Coccinella septempunctata*	36	10	1	3	8	52	34	8	28	0	3	183
*Coccinula quatuordecimpunctata* (L.)	0	0	0	0	0	0	1	0	0	0	0	1
*Exochomus quadripustulatus*	0	1	0	0	0	0	1	1	0	0	3	6
*Halizia sedecimguttata*	0	0	0	0	0	0	0	0	0	1	1	2
***Harmonia axyridis***	**0**	**1**	**5**	**5**	**4**	**15**	**4**	**7**	**3**	**18**	**20**	**82**
*Hippodamia variegata*	10	20	2	0	21	33	7	16	3	3	28	143
Platynaspis luteorubra*	5	0	1	0	0	0	2	10	0	1	0	19
*Propylea quatuordecimpunctata*	103	18	1	5	19	42	60	39	21	23	56	387
*Psyllobora vigintiduopunctata*	26	12	0	0	3	16	12	13	6	6	36	130
*Subcoccinella vigintiquatuoropunctata*	29	8	1	4	7	18	8	2	0	3	20	100
*Tytthaspis sedecimpunctata* (L.)	348	18	1	0	25	59	28	42	15	12	61	609
Total	559	94	15	27	89	237	157	138	76	72	235	1464

* In the field, *Platynaspis luteorubra* can be confused with Scymninae, a sub-family not covered in this study. Therefore, it cannot be ruled out that some specimens have been misidentified.

**Table 3:** Species and number of adult ladybirds collected at the five pine sites from 2007 to 2017. Numbers between parentheses indicate the number of collections. Ladybirds were not sampled in 2014.

	2007 (7)	2008 (8)	2009 (7)	2010 (7)	2011 (7)	2012 (7)	2013 (7)	2015 (7)	2016 (7)	2017 (7)	Total
*Adalia bipunctata*	1	0	2	0	0	0	0	0	0	0	3
*Adalia decempunctata*	2	2	1	0	0	2	2	0	4	11	24
*Anatis ocellata*	2	0	0	1	0	16	4	3	2	0	28
*Aphidecta obliterata*	1	4	0	2	0	3	0	0	10	0	20
*Chilocorus bipustulatus*	0	0	0	0	0	0	0	2	0	2	4
*Chilocorus renipustulatus*	0	0	0	0	0	0	0	0	0	1	1
*Coccinella quinquepunctata*	1	0	0	0	0	0	0	1	0	0	2
*Coccinella septempunctata*	5	0	0	18	39	15	4	5	1	15	102
*Exochomus quadripustulatus*	17	5	0	1	3	6	6	6	6	7	57
*Halizia sedecimguttata*	0	0	0	0	0	2	0	0	0	0	2
***Harmonia axyridis***	**2**	**6**	**0**	**37**	**27**	**8**	**2**	**3**	**16**	**25**	**126**
*Harmonia quadripunctata*	9	7	1	8	25	41	4	15	29	25	164
*Myrrha octodecimguttata* (L.)	0	0	0	0	0	3	0	0	6	0	9
*Myzia oblonguttata*	5	1	0	4	4	16	9	9	9	19	76
*Platynaspis luteorubra* *	0	0	0	0	0	0	2	0	0	0	2
*Propylea quatuordecimpunctata*	0	0	0	0	0	2	3	0	1	0	6
Total	45	25	4	71	98	114	36	44	84	105	623

* In the field, *Platynaspis luteorubra* can be confused with Scymninae, a sub-family not covered in this study. Therefore, it cannot be ruled out that some specimens have been misidentified.

**Table 4:** Species and number of adult ladybirds collected at the five spruce sites from 2007 to 2017. Numbers between parentheses indicate the number of collections. Ladybirds were not sampled in 2014.

	2007 (7)	2008 (8)	2009 (7)	2010 (7)	2011 (7)	2012 (7)	2013 (7)	2015 (7)	2016 (7)	2017 (7)	Total
*Adalia bipunctata*	4	0	0	0	0	0	0	0	0	0	4
*Adalia decempunctata*	74	4	0	6	0	0	0	2	0	1	87
*Anatis ocellata*	52	6	0	3	1	1	11	11	0	43	128
*Aphidecta obliterata*	671	366	99	126	123	137	254	237	121	284	2417
*Calvia quatuordecimguttata*	2	0	0	0	0	0	0	0	0	0	2
*Calvia decemguttata*	0	0	0	0	0	0	1	0	0	0	1
*Chilocorus renipustulatus*	0	0	0	0	3	0	0	0	0	0	3
*Coccinella septempunctata*	16	2	1	64	149	51	75	41	0	43	442
*Exochomus quadripustulatus*	0	0	0	3	0	1	2	1	0	4	11
*Halizia sedecimguttata*	2	0	0	1	0	1	0	0	0	0	4
***Harmonia axyridis***	**1**	**1**	**0**	**12**	**2**	**3**	**1**	**0**	**1**	**0**	**21**
*Harmonia quadripunctata*	0	0	0	0	0	1	0	0	0	0	1
*Myzia oblonguttata*	19	4	0	2	0	1	1	1	0	0	28
*Platynaspis luteorubra* *	0	0	0	0	0	0	1	0	0	0	1
*Propylea quatuordecimpunctata*	2	0	0	0	12	0	2	0	0	0	16
*Subcoccinella vigintiquatuorpunctata*	0	3	0	0	0	0	0	0	0	0	3
Total	843	386	100	217	290	196	348	293	122	375	3169

* In the field, *Platynaspis luteorubra* can be confused with Scymninae, a sub-family not covered in this study. Therefore, it cannot be ruled out that some specimens have been misidentified.

## Supplementary Materials

The following are available online at https://www.mdpi.com/2075-4450/11/12/883/s1. Table S1: Characteristics of the ladybird collection sites in the four habitats.
