# Supplementary material for: Long Term Monitoring in Switzerland Reveals That Adalia bipunctata Strongly Declines in Response to Harmonia axyridis Invasion
Source: Insects. 2020 Dec 12;11(12):883. doi: 10.3390/insects11120883 (PMC7764166; doi:10.3390/insects11120883)
Supplement: Supplementary file 1 [file insects-11-00883-s001.zip › Supplementary table S1.pdf]

# Long term monitoring in Switzerland reveals that Adalia bipunctata strongly declines in response to Harmonia axyridis invasion

**Marc Kenis, Saidou Nacambo, Johan Van Vlaenderen, Renate Zindel and René Eschen**

**Table S1.** Characteristics of the ladybird collection sites in the four habitats.

| Locality (canton)* | Years of collection | Habitat type | Main tree and shrub genera | Coordinates (°, N / E) | Elevation (m a.s.l.) |
|---|---|---|---|---|---|
| Bure (JU) | 2006-2017 | Broadleaved hedge | *Carpinus, Acer. Euonymus, Quercus, Prunus, Corylus, Cornus* | 47.4549 / 7.0038 | 540 |
| Cornol (JU) | 2006-2017 | Broadleaved hedge | *Corylus, Prunus, Salix, Populus, Acer, Cornus* | 47.4023 / 7.1493 | 520 |
| Bonfol (JU) | 2006-2017 | Broadleaved hedge | *Cornus, Prunus, Euonymus, Alnus, Fraxinus, Salix, Corylus, Quercus* | 47.4683 / 7.1565 | 440 |
| Chevenez (JU) | 2006-2017 | Broadleaved hedge | *Corylus, Prunus, Crataegus, Salix, Euonymus, Acer, Sambucus* | 47.3963 / 7.0151 | 470 |
| Lugnez (JU) | 2006-2017 | Broadleaved hedge | *Corylus, Euonymus, Prunus, Sambucus, Cornus* | 47.4883 / 7.0850 | 420 |
| Bourrignon (JU) | 2006-2017 | Broadleaved hedge | *Acer, Fraxinus, Fagus, Prunus, Corylus, Cornus, Tilia.* | 47.3825 / 7.2662 | 860 |
| Delémont (JU) | 2006-2017 | Broadleaved hedge | *Carpinus, Corylus, Crataegus, Salix,, Pyrus, Quercus* | 47.3574 / 7.3363 | 420 |
| Zwingen (BL) | 2006-2017 | Broadleaved hedge | *Aesculus, Euonymus, Salix, Fraxinus, Cornus, Juglans, Quercus, Populus* | 47.4344 / 7.5235 | 340 |
| Bubendorf (BL) | 2006-2017 | Broadleaved hedge | *Corylus, Crataegus, Acer, Prunus, Fraxinus, Carpinus* | 47.4369 / 7.7277 | 400 |
| Nenzlingen (BL) | 2006-2017 | Broadleaved hedge | *Prunus, Crataegus* | 47.4415 / 7.5627 | 340 |
| Aesch (BL) | 2006-2017 | Broadleaved hedge | *Corylus, Fagus, Cornus, Quercus, Acer* | 47.4680 / 7.5867 | 330 |
| Muttenz (BL) | 2006-2017 | Broadleaved hedge | *Corylus, Acer, Forsythia, Salix* | 47.5392 / 7.6354 | 280 |
| Augst (BL) | 2006-2017 | Broadleaved hedge | *Crataegus,Prunus, Cornus* | 47.5286 / 7.7280 | 300 |
| Ettingen (BL) | 2006-2017 | Broadleaved hedge | *Crataegus, Cornus, Lonicera, Sambucus, Prunus, Euonymus, Acer* | 47.4856 / 7.5609 | 350 |
| Riehen (BS) | 2006-2013 | Broadleaved hedge | *Syringa, Corylus, Acer, Rosa* | 47.5924 / 7.6451 | 270 |
| Füllinsdorf (BL) | 2015-2017 | Broadleaved hedge | *Acer, Corylus, Fagus, Crataegus, Prunus* | 47.5161 / 7.7332 | 400 |
| Bure (JU) | 2006-2017 | Meadow | n/a | 47.4546 / 7.0034 | 530 |
| Cornol (JU) | 2006-2017 | Meadow | n/a | 47.4023 / 7.1493 | 520 |
| Bonfol (JU) | 2006-2017 | Meadow | n/a | 47.4686 / 7.1564 | 440 |
| Chevenez (JU) | 2006-2017 | Meadow | n/a | 47.3960 / 7.0154 | 470 |
| Lugnez (JU) | 2006-2017 | Meadow | n/a | 47.4881 / 7.0846 | 420 |
| Develier (JU) | 2006-2017 | Meadow | n/a | 47.3568 / 7.3002 | 460 |
| Pleigne (JU) | 2006-2017 | Meadow | n/a | 47.4030 / 7.2943 | 790 |
| Zwingen (BL) | 2006-2017 | Meadow | n/a | 47.4346 / 7.5233 | 340 |
| Nenzlingen (BL) | 2006-2017 | Meadow | n/a | 47.4416 / 7.5625 | 340 |

| | | | | | |
|---|---|---|---|---|---|
| Muttenz (BL) | 2006-2017 | Meadow | n/a | 47.5392 / 7.6354 | 280 |
| Rheinfelden (AG) | 2006-2013 | Meadow | n/a | 47.5459 / 7.7594 | 280 |
| Bettingen (BS) | 2006-2013 | Meadow | n/a | 47.5118 / 7.6582 | 360 |
| Riehen (BS) | 2006-2013 | Meadow | n/a | 47.5921 / 7.6454 | 270 |
| Biel-Benken (BL) | 2006-2013 | Meadow | n/a | 47.5081 / 7.5358 | 330 |
| Zunzgen (BL) | 2006-2011 | Meadow | n/a | 47.4425 / 7.8085 | 410 |
| Bubendorf (BL) | 2012-2017 | Meadow | n/a | 47.4363 / 7.7277 | 400 |
| Aesch (BL) | 2015-2017 | Meadow | n/a | 47.4677 / 7.5870 | 325 |
| Ettingen (BL) | 2015-2017 | Meadow | n/a | 47.4856 / 7.5609 | 350 |
| Augst (BL) | 2015-2017 | Meadow | n/a | 47.5285 / 7.7283 | 300 |
| Füllinsdorf (BL) | 2015-2017 | Meadow | n/a | 47.5161 / 7.7330 | 400 |
| Lajoux (JU) | 2007-2017 | Spruce | *Picea abies* | 47.2644 / 7.1080 | 1000 |
| Les Genevez (JU) | 2007-2017 | Spruce | *Picea abies* | 47.2497 / 7.1372 | 1050 |
| Le Bémont (JU) | 2007-2017 | Spruce | *Picea abies* | 47.2493 / 7.0629 | 1020 |
| Montfaucon (JU) | 2007-2017 | Spruce | *Picea abies* | 47.2776 / 7.0421 | 1000 |
| Develier (JU) | 2007-2017 | Spruce | *Picea abies* | 47.3697 / 7.2809 | 640 |
| Soyhières 1 (JU) | 2007-2017 | Pine | *Pinus sylvestris* | 47.3914 / 7.3839 | 400 |
| Soyhières 2 (JU) | 2007-2017 | Pine | *Pinus sylvestris* | 47.3892 / 7.3695 | 400 |
| Liesberg (BL) | 2007-2017 | Pine | *Pinus sylvestris* | 47.3953 / 7.4323 | 400 |
| Vicques (JU) | 2007-2017 | Pine | *Pinus sylvestris* | 47.3674 / 7.4353 | 570 |
| St-Brais (JU) | 2007-2017 | Pine | *Pinus sylvestris* | 47.3091 / 7.1220 | 950 |

*Canton: BL: AG: Aargau; Basel-Landschaft; BS: Basel-Stadt; JU: Jura.
